# Supplementary material for: The Construction and Exploration of a Comprehensive MicroRNA Centered Regulatory Network in Foxtail Millet (Setaria italica L.)
Source: Front Plant Sci. 2022 May 6;13:848474. doi: 10.3389/fpls.2022.848474 (PMC9121102; doi:10.3389/fpls.2022.848474)
Supplement: Supplementary file 4 [file Data_Sheet_4.zip › Sit-miRN3021_Seita.7G298100.1_1301_TPlot.pdf]

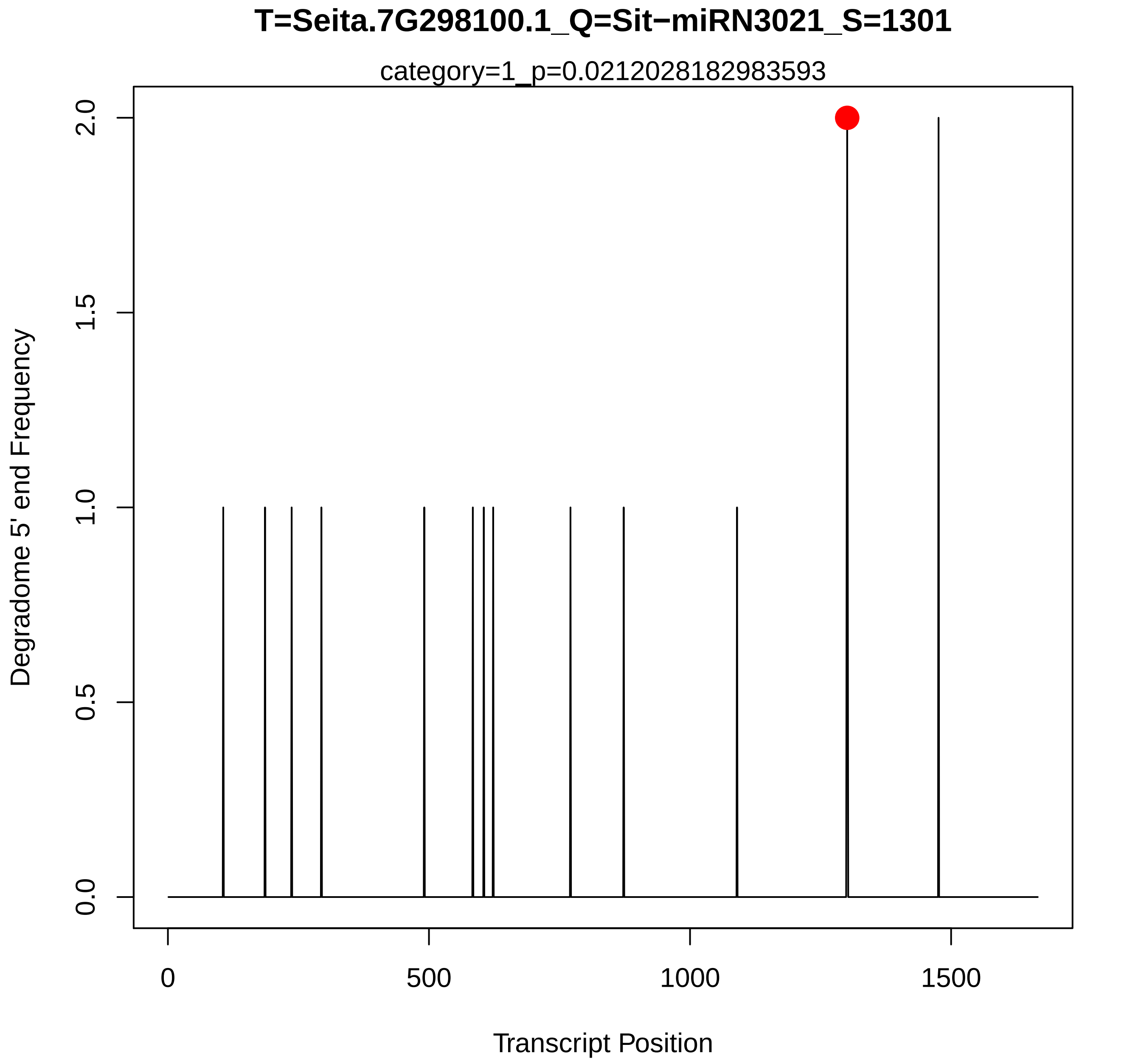
T=Seita.7G298100.1_Q=Sit−miRN3021_S=1301
category=1_p=0.0212028182983593
Degradome 5' end Frequency
0.0
0.5
1.0
1.5
2.0
0
500
1000
1500
Transcript Position
